# Maternal Habitual Midday Napping Duration and Frequency are Associated with High Birthweight

**DOI:** 10.1038/s41598-017-09683-3

**Published:** 2017-09-05

**Authors:** Xiaoxuan Zheng, Lina Zhang, Lijun Shen, Lulu Song, Hui Li, Bingqing Liu, Yuanyuan Li, Wei Xia, Bin Zhang, Shunqing Xu, Youjie Wang

**Affiliations:** 1 Department of Maternal and Child Health, School of Public Health, Tongji Medical College, Huazhong University of Science and Technology, Wuhan, Hubei Province People’s Republic of China; 2 Key Laboratory of Environment and Health, Ministry of Education & Ministry of Environmental Protection, and State Key Laboratory of Environmental Health, School of Public Health, Tongji Medical College, Huazhong University of Science and Technology, Wuhan, Hubei Province People’s Republic of China; 3 Wuhan Medical & Healthcare Center for Women and Children, Wuhan, Hubei Province People’s Republic of China

## Abstract

Habitual midday napping is a common habit in China, especially for pregnant women. The purpose of this study was to examine whether duration and frequency of maternal habitual midday napping were associated with high birthweight (HBW). A total of 10,482 participants from Healthy Baby Cohort were include in our analysis. The information of the mothers and their infants were abstracted from medical records, or obtained from questionnaire. Logistic regression models were used to calculate the odds ratios (ORs) and 95% confidence intervals (CIs) of habitual midday napping duration and frequency with HBW. Of the participants, 8,705 (83.0%) reported having habitual midday napping. Duration and frequency of napping had a positive association with HBW without adjustment. After controlling for potential confounders, increasing risk of HBW was observed in participants who napped 1.5–2 hours (OR, 1.50, 95% CI, 1.14, 1.98), and ≥2 hours (OR, 1.35, 95% CI, 1.03, 1.78) compared with no habitual midday napping. Participants who took naps ≥5 days/week had a higher risk of HBW (OR, 1.37, 95% CI, 1.07, 1.77) compared with the women without naps. This suggests that longer (≥1.5 hours) and more frequent (≥5 days/week) maternal habitual midday napping were associated with an increased risk of HBW.

## Introduction

Habitual midday napping is common practice in China^1^, typically taken after lunch and ranging from several minutes to several hours. Habitual midday napping is a cross-culture phenomenon that occurs across a person’s lifespan^1–3^. In Chinses society, midday napping is regarded as a health-promoting habit because of a postulated “stress relief” mechanism^4^. However, it also has been regarded as an early indicator of a variety of chronic conditions, such as diabetes and obesity^5, 6^, and associated with higher morbidity and mortality^4, 7^.

Birthweight is a prenatal factor that has important effects on future health status^8^, and has long-term effect on organ and mental development^9^. High birthweight (HBW) is defined as a birthweight more than 4000 g, which is common in obstetrics and poses an increased risk of complications for both mothers and newborns^10, 11^. High birthweight is not only a cause of cesarean delivery, shoulder dystocia and perinatal trauma^12^, but also a risk factor for offspring overweight/obesity^13^, and developing diabetes, and cardiometabolic disease in adulthood^14–16^.

Several maternal and fetal factors have been reported to be associated with birthweight, especially maternal lifestyle during pregnancy like physical activity, psychosocial stress, etc.^17^. Epidemiological studies have suggested an association between maternal night sleep practice and birthweight^18^. However, the relationship between habitual midday napping and birthweight has not been explored. Habitual midday napping is practiced by all ages, beginning as a childhood habit in China^5^. A clearer picture of the health impact of maternal habitual midday napping might thus be visible in such a setting.

The purpose of this study was to investigate the relationship between maternal habitual midday napping duration and frequency and the prevalence of HBW.

## Results

The descriptive characteristics of 10,482 participants are shown in Table 1, stratified by HBW. Maternal age, education, employment during pregnancy, passive smoking status, parity, physical activity, night sleep duration and household income per year were similar between HBW and normal birthweight. Participants who had higher pre-pregnancy body mass index (BMI), delivered a baby boy or were diagnosed with gestational diabetes mellitus (GDM) were more likely to have a HBW baby (*P* < 0.05).

**Table 1:** The characteristics of study participants of 10,482 women by HBW.

	Normal birthweight	High birthweight		
	n = 9,868	n = 614	χ^2^/F	*P*
Maternal age(year)(mean ± SD)	28.20 ± 3.66	28.49 ± 3.65	1.104	0.316*
Education (%)				
More than high school	1,361(94.3%)	83(5.7%)		
High school	1,847(94.7%)	104(5.3%)	1.392	0.499^+^
Less than high school	6,655(94.0%)	427(6.0%)		
Missing	5 (100.0%)			
Household income (Yuan/per year) (%)				
<50000 ($7350)	4,096(94.2%)	252(5.8%)		
50000–($7350–)	3,895(94.3%)	236(5.7%)	0.800	0.670^+^
≥100000($14700)	1,714(93.7%)	115(6.3%)		
Missing	163(93.7%)	11(6.3%)		
Employment during pregnancy (%)				
Employed	4,630(93.8%)	305(6.2%)	1.706	0.191^+^
Unemployed	5,229(94.4%)	309(5.6%)		
Missing	9(100.0%)			
BMI before pregnancy (kg/m^2^) (%)				
<18.5	2,298(97.0%)	72(3.0%)		
18.5–	6,584(94.2%)	409(5.8%)	124.967	<0.0001^+^
24–28	847(89.3%)	101(10.7%)		
≥28	139(91.3%)	32(18.7%)		
Passive smoking (%)				
Yes	8,650(94.0%)	550(6.0%)	1.984	0.159^+^
No	1,218(95.0)	64(5.0%)		
Parity (%)				
0	8,426(94.1%)	529(5.9%)	0.275	0.600^+^
≥1	1,442(94.4%)	85(5.6%)		
Infant sex (%)				
Boy	5,117(92.6%)	406(7.4%)	47.215	<0.0001^+^
Girl	4,751(95.8%)	208(4.2%)		
Gestational age (week)(%)				
<37	350(100.0%)	0(0%)		
37–	9,500(93.9%)	613(6.1%)	38.316	<0.0001^#^
≥42	18(94.7%)	1(5.3%)		
Physical activity (%)				
Never	1,065(95.1%)	55(4.9%)		
Not everyday	1,742(93.2%)	128(6.8%)	5.317	0.070^+^
Everyday	6,995(94.3%)	426(5.7%)		
Missing	66(93.0%)	5(7.0%)		
GDM (%)				
Yes	770 (89.1%)	94 (10.9%)	43.064	<0.0001^+^
No	9,098(94.6%)	520(5.4%)		
Night sleep duration (hours) (%)				
<7	93(96.9%)	3(3.1%)		
7–	536(93.7%)	36(6.3%)	1.497	0.473^+^
≥8	9,192(94.1%)	572(5.9%)		
Missing	47(94.0%)	3(6.0%)		

Note Abbreviations: BMI, body mass index; GDM, gestational diabetes mellitus.

Data are mean ± SD or n (%).

^*^student *t-test* for normally distributed data;

^+^χ^2^ test for categorical data;

^#^Fisher’s exact test for categorical data.

The unadjusted and adjusted odds ratios (ORs) and 95% confidence intervals (CIs) for HBW according to habitual midday napping duration are shown in Table 2. Relatively few participants reported no habitual midday napping (17.0%), while 30.7% reported more than 2 hours of habitual midday napping. Longer habitual midday napping was significantly associated with an increased risk of HBW. Without any adjustment, the ORs (95% CIs) for HBW comparing no habitual midday napping (reference) were 1.36 (95% CI, 1.03, 1.79) for the participants who took habitual midday naps less than 1.5 hours, 1.47 (95% CI, 1.12, 1.92) for 1.5–2 hours, and 1.33 (95% CI, 1.02, 1.74) for more than 2 hours. After adjustment for maternal age, infant sex, gestational age, pre-pregnancy BMI, yearly household income, parity, education, passive smoking, and GDM, the ORs (95% CIs) were 1.50 (95% CI, 1.14, 1.98) and 1.35 (95% CI, 1.03, 1.78) for habitual midday napping for 1.5–2 hours and ≥2 hours, respectively, compared with the participants who never had habitual midday naps.

**Table 2:** Unadjusted and adjusted odd ratios (95%CI) for HBW by maternal habitual midday napping duration.

Habitual midday napping duration	Number	Case	OR^a^(95%CI)	OR^b^(95%CI)
Never	1,777	80(4.5%)	1.00 (reference)	1.00 (reference)
<1.5 hour	2,604	157(6.0%)	1.36(1.03, 1.79)	1.28(0.96, 1.71)
1.5–2 hours	2,887	187(6.5%)	1.47(1.13, 1.92)	1.50(1.14, 1.98)
≥2 hours	3,214	190(5.9%)	1.33(1.02, 1.74)	1.35(1.03, 1.78)

^a^Unadjusted odds ratio.

^b^Adjusted for maternal age, infant sex, gestational age, pre-pregnancy BMI, yearly household income, parity, education, GDM and passive smoking.

As shown in Table 3, 64.0% participants reported that they had habitual midday naps more than 5 days per week; 12.1% took such naps 3 to 4 days and 7.0% took them 1–2 days per week. The results of the unadjusted model indicated that more frequent habitual midday napping (≥5 days/week) was related to an increased risk for HBW (OR, 1.39, 95% CI, 1.09, 1.78). The adjusted model controlled for potential confounders (maternal age, infant sex, gestational age, pre-pregnancy BMI, yearly household income, parity, education, passive smoking, and GDM) also showed similar results (OR, 1.37, 95% CI, 1.07, 1.77). The rate of HBW for the participants who had habitual midday napping 1–2 and 3–4 days/week were numerically but not significantly higher compared with the participants who never had.

**Table 3:** Unadjusted and adjusted odd ratios (95%CI) for HBW by maternal habitual midday napping frequency.

Habitual midday napping frequency	Number	Case	OR^a^(95%CI)	OR^b^(95%CI)
Never	1,777	80(4.5%)	1.00 (reference)	1.00 (reference)
1–2 days per week	729	45(6.2%)	1.40(0.96, 2.03)	1.45(0.99, 2.12)
3–4 days per week	1266	76(6.0%)	1.36(0.98, 1.87)	1.37(0.98, 1.90)
5–7 days per week	6,710	413(6.2%)	1.39(1.09, 1.78)	1.37(1.07, 1.77)

^a^Unadjusted odds ratio.

^b^Adjusted for maternal age, infant sex, gestational age, pre-pregnancy BMI, yearly household income, parity, education, GDM and passive smoking.

## Discussion

In the present study, we found that longer (≥1.5 hours) and more frequent (≥5 days/week) maternal habitual midday napping was associated with an increased risk of HBW. To the best of our knowledge, this is the first study to investigate the association of maternal habitual midday napping with HBW in China.

No previous studies have assessed the association between maternal habitual midday napping and birthweight. However, maternal sleep pattern have been reported to be related to fetal growth. Previous studies had reported that maternal short nighttime sleep is associated with a higher risk of preterm birth and large-for-gestational-age neonates^19, 20^. Maternal sleep practice (including snoring and supine sleep) also has been involved in adverse pregnancy outcomes such as low birthweight and stillbirth^18^.

The mechanism underlying the association between habitual midday napping duration and HBW is unclear. Habitual midday napping could affect several potential inter-related sleep and circadian mechanisms^21^, which might influence multiple physiological functions, especially metabolic and endocrine functions^22^. Excessive habitual midday napping enhances the release of endogenous glucocorticoid secretion, which may increase the body’s resistance to insulin^23^. Maternal insulin resistance could increase glucose availability to the fetus and the risk of fetal overgrowth^24, 25^. Xu *et al*. reported that regardless of night sleep duration, long habitual midday napping (>1 h) was associated with high risk of diabetes^26^. Similar results were also found in pregnant women^27^. Izci *et al*. observed a positive association (OR 1.64; 95% CI, 1.00–2.68) between the high glucose challenge test and longer self-reported habitual midday napping duration in early pregnancy in a case-control study^27^. Moreover, habitual midday napping might influence the sleep/wake cycles, cause excessive hormone fluctuation, and disrupt the regulation of glucose metabolism and appetite^28^; and above all could contribute to the relationship between maternal habitual midday napping and HBW^12^.

Evidence from epidemiological studies indicated that longer habitual midday napping was independently associated with obesity^29^, which is also a strong and independent risk factor of HBW^30^. Longer habitual midday napping may reduce physical activity^31^, thermogenesis and daily energy consumption, which is an alternative mechanism that could cause obesity^32^. Additionally the obese mothers had nearly double the risk of HBW compared with that of the normal weight mothers^33^. In a population-based study, Shi *et al*. found that maternal overweight was significantly associated with HBW after adjusting for maternal age and gestational weeks^34^.

There are several limitations in the present study that deserve acknowledgement. First, the duration and frequency of habitual midday napping were self-reported and not measured in our study. Further studies should use actigraphy or other objective measures. Second, although longer and more frequent habitual midday napping was shown to be associated with a higher risk of HBW, causal and temporal sequence association could not be inferred due to the cross-sectional design. Third, we only asked the participants about their habitual midday napping for one month before delivery, not for the entire pregnancy. Thus, the conclusion of our study may not be generalized to the earlier pregnancy period. Finally, data about confounders affecting habitual midday napping and HBW (such as dietary, psychological factors) were not measured in the present study, which might result in residual confounding.

In summary, these results suggest that women taking long and regular habitual midday naps have a higher risk for HBW, but the mechanism underlying this association is uncertain. These findings could have clinical implications for possible preventative steps. Encouraging pregnant women to have a rational duration and frequency of habitual midday napping might be an intervention to reduce the risk of HBW.

## Methods

### Study participants

This study used data from the prospective Healthy Baby Cohort (HBC). This ongoing cohort enrolls participants at Wuhan Medical & Healthcare Center for Women and Children in Hubei province, China. The participants had been asked to participate in the cohort study and complete a face-to-face interview when they gave birth at the hospital. The recruitment started in November 2012. The eligibility criteria for participants are as follows: 1) residence in the study areas at the time of the recruitment period with an expectation to reside continually in these areas for the foreseeable future; and 2) ability to understand and complete the questionnaire.

Of the 11,311 eligible participants, we excluded women whose information on habitual midday napping or infant birthweight were missing (n = 373). We also excluded the subjects whose infant birthweight was less than 2500 g (n = 456). The final study sample included 10, 482 participants.

### Ethics Statement

Signed informed consent was obtained from all participants, and the study was approved by the Medical Ethics Committee of the School of Public Health, Tongji Medical College, Huazhong University of Science and Technology, and the Women & Children Medical and Healthcare Centre of Wuhan. All the methods in the present study were carried out in accordance with the approved guidelines.

### Data collection

#### Assessment of habitual midday napping duration and frequency

Habitual midday napping frequency was assessed by asking “How many times a week did you take a midday nap over the past month?” Those who had a habit of taking a midday nap were further asked about duration. We categorized habitual midday napping frequency into 4 groups: never, 1–2 times per week, 3–4 times per week, and 5–7 times per week. Habitual midday napping duration was divided into never, <1.5 hours, 1.5–2 hours, and ≥2 hours.

#### Assessment of high birthweight

Infant birthweight was abstracted from medical records. Birthweight greater than 4000 g was defined as HBW.

#### Assessment of covariates

Demographic information on maternal age, education (e.g., less than high school, high school, more than high school), employment (e.g., employed or unemployed during pregnancy), and yearly household income (e.g., less than 50,000, 50,000–, ≥100,000 Yuan) was collected by face-to-face interview. Lifestyle information on night sleep duration (e.g., <7, 7–, ≥8 hours), passive smoking during pregnancy, and physical activity (e.g., never, not every day, every day) were also obtained by questionnaires. Passive smokers were defined as those exposed to passive smoking for more than 15 minutes per day during pregnancy. Information of parity, infant sex, gestational age and diagnosis of GDM were abstracted from medical records. Pre-pregnancy BMI was calculated using the self-reported weight (kg) divided by measured height (m^2^).

### Statistical analysis

Categorical data were summarized as proportion (%) and normally distributed data were summarized as mean ± SD. Differences between groups were analyzed with Student’s *t-test* for normally distributed data and χ^2^ tests for categorical variables. We used HBW and normal birthweight as the dependent variables in the multivariate logistic regression-model, and calculated the ORs and 95% CIs. We used women who never took habitual midday naps as the reference category, and examined the association between habitual midday napping duration and frequency with HBW. Covariates included maternal age, gestational age, infant sex, pre-pregnancy BMI, household income, parity, education, GDM and passive smoking during pregnancy. After diagnosis of collinearity, all of these covariates were adequate to adjust in the logistic model (tolerance: 0.754–0.998). All statistical analyses were conducted using SPSS software (version 17.0). Associations were considered significant at *P* < 0.05.
